# Enucleation of the *C. elegans* embryo revealed dynein-dependent spacing between microtubule asters

**DOI:** 10.26508/lsa.202302427

**Published:** 2023-11-06

**Authors:** Ken Fujii, Tomo Kondo, Akatsuki Kimura

**Affiliations:** 1 Department of Genetics, School of Life Science, Sokendai (Graduate University for Advanced Studies ) Mishima, Japan; 2 Cell Architecture Laboratory, National Institute of Genetics , Mishima, Japan

## Abstract

The intracellular positioning of the centrosome, a major microtubule-organizing center, is important for cellular functions. One of the features of centrosome positioning is the spacing between centrosomes; however, the underlying mechanisms are not fully understood. To characterize the spacing activity in *Caenorhabditis elegans* embryos, a genetic setup was developed to produce enucleated embryos. The centrosome was duplicated multiple times in the enucleated embryo, which enabled us to characterize the chromosome-independent spacing activity between sister and non-sister centrosome pairs. We found that the timely spacing depended on cytoplasmic dynein, and we propose a stoichiometric model of cortical and cytoplasmic pulling forces for the spacing between centrosomes. We also observed dynein-independent but non-muscle myosin II-dependent movement of centrosomes in the later cell cycle phase. The spacing mechanisms revealed in this study are expected to function between centrosomes in general, regardless of the presence of a chromosome/nucleus between them, including centrosome separation and spindle elongation.

## Abstract (precis)

Using a genetic trick to produce enucleated *C. elegans* embryos, the authors revealed how the microtubule asters take space between each other inside the cell.

## Introduction

Centrosomes are the major microtubule-organizing centers in animal cells (Azimzadeh & Bornens, 2007). Centrosomes cooperate with microtubules to play important roles in intracellular transport and cell division (O’Connell, 2000; Meraldi, 2016). The positioning of centrosomes in cells is important for various cellular functions (Tang & Marshall, 2012; Elric & Etienne-Manneville, 2014). In interphase, the centrosomes tended to be located in the cell center. Because the centrosome is associated with the nucleus, this position is important for positioning the nucleus at the center of the cell (Silkworth et al, 2012). During mitosis, the two centrosomes become the poles of the mitotic spindle, and their positions define the direction and asymmetry of cell division (Grill et al, 2001).

The position of centrosomes is controlled by the forces generated by microtubules and motor proteins associated with microtubules. Using microtubules and motor proteins, the centrosome interacts with various structures such as the cell cortex, cytoplasmic vesicles, nucleus, and chromosomes (Dogterom & Yurke, 1997; Gönczy et al, 1999; Malone et al, 2003; Grill & Hyman, 2005; Mogilner et al, 2006; Kimura & Kimura, 2011). In addition to these intracellular structures, centrosomes also interact with each other to position themselves. These two centrosomes, which share a common cytoplasm, appear to repel each other. This repulsive movement is observed along the nuclear surface, known as centrosome separation, and within the mitotic spindle. In addition, centrosomes take space between each other independent of sliding along the nuclear surface or spindle formation. In this study, this nucleus and spindle-independent activity is defined as the “spacing” activity of the centrosomes. In a classic experiment demonstrating the formation of a cell division furrow between non-sister pairs of centrosomes (i.e., the “Rappaport furrow”), these pairs took space between each other (Rappaport, 1961; Oegema & Mitchison, 1997). In *Drosophila* syncytium cells, nuclei and spindles are positioned at a certain spacing (Kanesaki et al, 2011; Telley et al, 2012; de-Carvalho et al, 2022). Similar spacing was observed in the oocytes of drug-treated marine ascidians (Khetan et al, 2021) and in the self-organized cell-like organization of *Xenopus* egg extracts (Cheng & Ferrell, 2019). These observations suggest a repulsive interaction between centrosomes.

The sliding of plus-end–directed motors between antiparallel microtubules that elongate from each pair of centrosomes is the only mechanism proposed for the centrosome spacing (Baker et al, 1993). This model is analogous to the mechanism underlying the spindle pole separation in *Drosophila* anaphase B (Brust-Mascher et al, 2004). In support of this model, bipolar kinesin-5 (Klp61F), a microtubule-bundling protein PRC1 (Fascetto/Feo), and kinesin-4 (Klp3A) were localized to slide antiparallel microtubules between centrosomes in the *Drosophila* syncytium (Deshpande et al, 2022). PRC1 (Prc1E) and kinesin-4 (Kif4A) have been shown to separate centrosomes from *Xenopus* egg extracts (Nguyen et al, 2014, 2018). In summary, previous studies on the spacing activity between centrosomes have focused on plus-end–directed motor sliding along antiparallel microtubules. Other mechanisms underlying the spacing between centrosomes are unknown.

Here, we aimed to reveal a novel mechanism for the spacing between centrosomes. The *Caenorhabditis elegans* embryo is a well-studied model for centrosome biology. Interestingly, unlike humans, *Xenopus*, and *Drosophila*, *C. elegans* orthologs of proteins involved in the sliding of antiparallel microtubules (BMK-1 [kinesin-5 ortholog], SPD-1 [PRC1 ortholog], and KLP-19 [kinesin-4]) are not required for mitotic spindle elongation in the embryo (Powers et al, 2004; Saunders et al, 2007; Lee et al, 2015). The investigation of the spacing activity of the centrosomes in the *C. elegans* embryo should have an impact on *C. elegans* biology and on other species because kinesin-independent spacing has been suggested in other species (Donoughe et al, 2022).

It is challenging to characterize centrosome spacing activity, which is independent of the nucleus and spindle, in the *C. elegans* embryo. Centrosomes in *C. elegans* embryos are always associated with the nucleus or spindle, and the embryonic cells do not form syncytia. Inactivation of the *zyg-12* gene offers some information on centrosome spacing, independent of the nucleus, because this gene encodes a KASH domain protein that is essential for the association between the centrosome and the nucleus (Malone et al, 2003). In *zyg-12*-impaired cells, centrosomes move even when they are not attached to the nucleus until they are incorporated into the mitotic spindle. Upon inactivation of *zyg-12*, the two centrosomes in the one-cell stage embryo separate, indicating that spacing activity is independent of the nucleus. Centrosome separation is also impaired by the RNAi of genes involved in the cortical pulling force, suggesting that this force affects spacing (De Simone et al, 2016). However, it is unclear how the two centrosomes move in opposite directions instead of being pulled toward the same cortical region. It has been proposed that the cytoplasmic flow contributes to this process (De Simone et al, 2016). However, this model does not ensure that two centrosomes move in opposite directions. In addition, cytoplasmic flow occurred only at the one-cell stage. Therefore, this mechanism cannot be considered a general mechanism for centrosome spacing. *zyg-12* affects the interaction between the nucleus and centrosome, but not spindle formation. Therefore, a spacing mechanism independent of the spindle could not be identified in the *zyg-12*-impaired cells. Spacing activity, which is independent of the nuclei and spindles and is generally involved in multiple stages of embryogenesis, was expected but uncharacterized in the *C. elegans* embryo.

## Results

### Establishment of enucleated *C. elegans* embryos by genetic manipulation

To characterize the spacing activity between the two centrosomes, an experiment was designed to remove the chromosomes from the *C. elegans* embryo (“enucleated embryo”). Enucleated *C. elegans* embryos were produced in classic experiments by Schierenberg and Wood, where the nuclei are removed by penetration of the eggshell using laser microsurgery, followed by pressing the cytoplasm to push the nucleus out of the eggshell (Schierenberg & Wood, 1985). This method often removes the centrosome together with the nucleus and is unsuitable for analyzing centrosome behavior.

**Figure S1.**
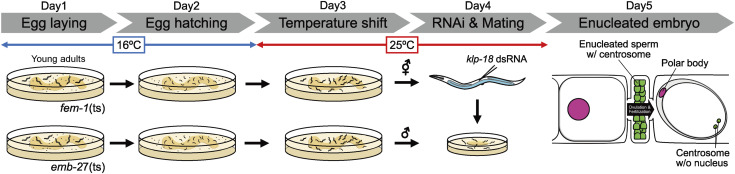
The procedure of enucleation of the *C. elegans* embryo. Related to Fig 1. Schematic of the enucleation procedure. For details, see the Materials and Methods section. Day 1: young adults were transferred to fresh culture plates to lay eggs. Day 2: the adults were removed from the plates. Day 3: the plates were moved from 16°C to 25°C. Day 4: hermaphrodites were injected with *klp-18* dsRNA. After injection, hermaphrodites were cultured on a smaller plate containing males. Day 5: hermaphrodites were dissected and observed under a fluorescence microscope.

To create an enucleated embryo with centrosomes, paternal and maternal chromosomes were removed using *emb-27* mutant sperm (Sadler & Shakes, 2000), and by knocking down the *klp-18* gene (Segbert et al, 2003) (Fig S1). *emb-27* encodes a subunit of an anaphase-promoting complex. This mutation causes chromosome segregation defects and produces centriole-containing fertilization-competent enucleated sperm (Sadler & Shakes, 2000; Kondo & Kimura, 2018 *Preprint*). *klp-18*, a member of the kinesin family, is required for oocyte meiosis. The *klp-18* knockdown oocyte occasionally extrudes all chromosomes into the polar body, resulting in embryos without maternal chromosomes. By mating the worms with *emb-27* mutant sperms and *klp-18* knockdown oocytes, we expected to obtain enucleated embryos to characterize the spacing activity of the centrosomes independent of the nucleus and spindle.

**Figure 1.**
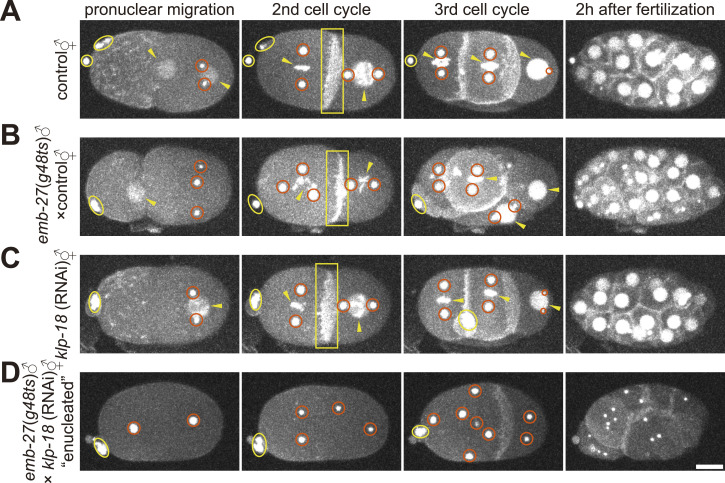
Establishment of enucleated *C. elegans* embryos by genetic manipulation. **(A, B, C, D)** γ-tubulin (centrosome), histone H2B (chromosome), and PH^PLCδ1^ (cell membrane) were labeled with GFP. Red circles indicate centrosomes. Yellow arrowheads indicate the pronucleus, nucleus or chromosome. The yellow ovals indicate the polar body. Yellow squares indicate the cell membranes. z-maximum projections. Scale bar, 10 μm. **(A)** A time lapse-imaging series of an embryo of the control strain (CAL0181) grown at 16°C, with imaging at 18–22°C. The reproducibility of the observations was confirmed (n = 5). **(B)** An embryo from a hermaphrodite of CAL0181 strain mated with males of CAL0051 strain. Both strains were grown at 25°C, with imaging at 18–22°C (n = 5). The embryo of this figure initially possessed three centrosomes at the one-cell stage. **(C)** An embryo of the hermaphrodite of CAL0181 with *klp-18* (RNAi) grown at 25°C after injection, with imaging at 18–22°C (n = 5). **(D)** An embryo of the hermaphrodite CAL0181 with *klp-18* (RNAi) was mated with males of the CAL0051 strain. Both strains were grown at 25°C, with imaging at 18–22°C (n = 7).

In this study, *C. elegans* strains were used in which the centrosomes (γ-tubulin), chromosomes (histone H2B), and cell membranes (PH^PLCδ1^) were visualized using GFP (Fig 1 and Table S1). In control one-cell stage embryos, sperm- and oocyte-derived pronuclei appeared after fertilization (Fig 1A and Video 1 [left]). The two centrosomes associated with the sperm pronucleus move toward the cell center and meet the oocyte pronucleus before the first cytokinesis. When *emb-27*(*g48*ts) males were mated with control hermaphrodites, the sperm pronucleus was absent, as previously reported (Sadler & Shakes, 2000) (Fig 1B and Video 1 [middle]). The centrosomes migrate toward the cell center and meet the oocyte pronucleus. The *emb-27* mutants affected the number of centrosomes supplied by the sperm (Kondo & Kimura, 2018 *Preprint*, 2019). Consequently, the presence of 1–4 centrosomes was observed in the one-cell stage *emb-27* mutant and in the enucleated embryo. Oocyte pronuclei were not detected in *klp-18* (RNAi) embryos, as previously reported (Segbert et al, 2003) (Fig 1C and Video 1 [right]). We designed an experiment to obtain embryos without chromosomes by mating *emb-27*(*g48*ts) males with *klp-18* (RNAi) hermaphrodites. Enucleated embryos were successfully obtained using this experimental setup (Fig 1D and Video 2). No sperm- or oocyte-derived pronuclei or chromosomes were detected in the embryonic cells at subsequent stages. Chromosomal signals from the polar bodies were detected outside the cytoplasm.

Table S1 Strains used in this study.

Video 1 Centrosome movement and cell division in *C. elegans* embryos. (Left) Time-lapse movie corresponding to Fig 1A (control embryo). (Middle) Time-lapse movie corresponding to Fig 1B (*emb-27*(*g48ts*) mutant embryo). (Right) Time-lapse movie corresponding to Fig 1C (*klp-18* (*RNAi*) embryo). Time-lapse movie of *C. elegans* embryos expressing *GFP*::*histone H2B*, *tbg-1*::*GFP*, *GFP*::*PH*^*PLC1δ1*^. In the first five frames, yellow arrows indicate centrosomes, yellow arrowheads indicate pronuclei, and yellow circles indicate polar bodies. A movie of the 2-h imaging is shown. z-maximum projections. Time indicated in min. Time 0 was when the imaging started. The time interval between the measurements was 1 min. Scale bar, 10 μm. Download video

Video 2 Centrosome movement and cell division in *emb-27*(*g48ts*) mutant and *klp-18* (RNAi) *C. elegans* embryos (enucleated embryos). Time-lapse movie corresponding to Fig 1D. Imaging conditions were the same as those used in Video 1. Download video

Centrosomes moved dynamically in enucleated embryos, which was the main topic of this study. This indicates that centrosomes can move without requiring nuclei or chromosomes. Interestingly, the centrosomes duplicated periodically for multiple rounds, which appeared to correspond to the cell cycle. Cytokinesis was impaired for at least several cell cycles (Fig 1D), possibly because of chromosomal loss (Bringmann & Hyman, 2005). Most importantly, in this study, the positions of these centrosomes did not overlap, but were spread throughout the cell (Fig 1D), suggesting the existence of spacing activity. Therefore, the enucleated *C. elegans* embryo is suitable for analyzing the centrosome-spacing mechanism, which is independent of the nucleus and spindles. In summary, sister and non-sister centrosomes share a common cytoplasm and move dynamically in an enucleated embryo.

### A repulsive spacing between sister and non-sister centrosomes was observed in the enucleated embryo

**Figure 2.**
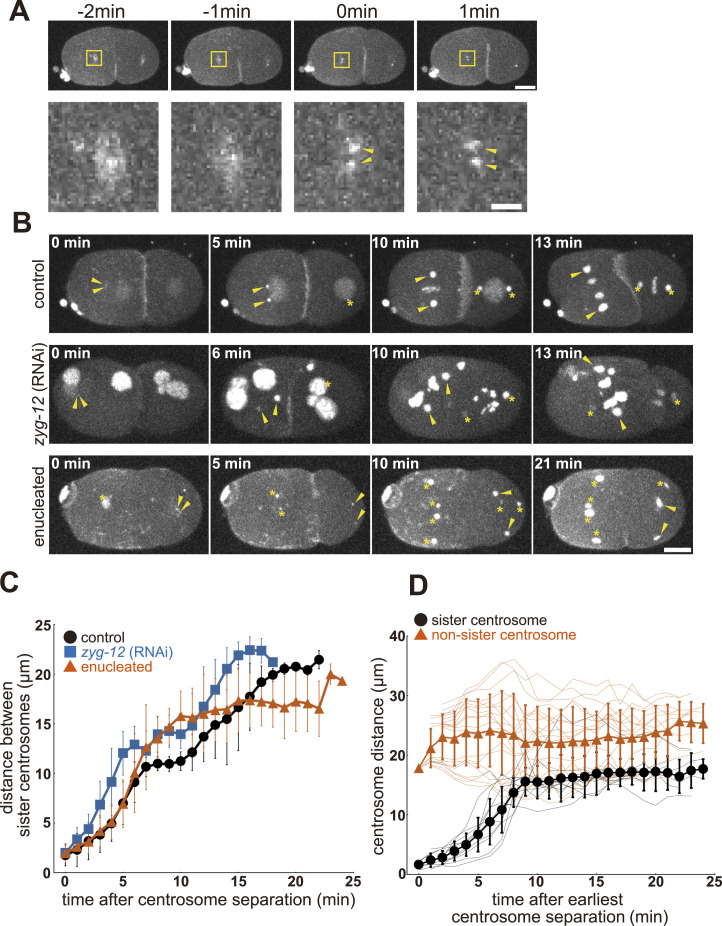
Characterization of centrosome dynamics during the two-cell stage. **(A)** The definition of time zero of the two-cell stage. Representative time-series images (upper) and the enlarged images of the yellow box (lower) of an enucleated embryo. The time point when we detected two discrete spots in the cloud of the γ-tubulin::GFP signal was defined as time zero. The yellow arrowheads indicate two discrete spots of centrosomes. z-maximum projections. Scale bars represent 10 μm for upper panels and 2 μm for lower panels. **(B)** A time-lapse imaging series of an embryo of the control strain (CAL0181), a DE90 strain with *zyg-12* (RNAi) embryo, and an enucleated embryo in the two-cell stage. The yellow arrowheads indicate a pair of sister centrosomes. The asterisks indicate the other centrosomes in the images. z-maximum projections. Scale bar, 10 μm. **(C)** The quantification of the distance between sister centrosomes. The mean and SD are shown with the symbol and the error bar, respectively. Black circle, control embryos (8 sister pairs from 5 embryos). Blue square, *zyg-12* (RNAi) embryos (7 sister pairs from 5 embryos). Red triangle, enucleated embryos (10 sister pairs from 5 embryos). **(D)** Distance between the sister- and non-sister pairs of centrosomes in the enucleated embryo. The distances between the non-sister pairs are calculated for all possible pairs of the non-sisters. Individual samples are shown with thin lines. To compare the sister and non-sister pairs, the time after the earliest centrosome separation of the cell is indicated in the horizontal axis, which is slightly different from the time in (C). The mean and SD are shown with the symbol and the error bar, respectively. Black circle, sister pairs (10 pairs from 5 embryos). Red triangle, non-sister pairs (20 pairs from 5 embryos).

To characterize the force acting between the centrosomes, the change in distance between the centrosomes over time was quantified. In the present study, we focused on the time window corresponding to the two-cell stage in control embryos with nuclei (Fig 2A and B). In the enucleated embryo, the cytokinesis failed; therefore, the cytoplasm did not divide into two in the “two-cell stage.” At this stage, four (or more, depending on the number of centrosomes in the one-cell stage, as explained in the previous section) centrosomes of the two sister and non-sister pairs coexist in the common cytoplasm. We focused on this stage because it was the earliest at which potential interactions between non-sister pairs of centrosomes could be tracked. We set the time zero of the time window when we first detected two discrete centrosome (γ-tubulin::GFP) spots for the sister pair after the second centrosome duplication (Fig 2A). The time window ended when the signal of the spot became too weak to be identified or when the spot was duplicated into two in the subsequent round of centrosome duplication.

The distances between sister pairs of centrosomes in enucleated embryos, controls (i.e., embryos with nuclei), and *zyg-12* (RNAi) embryos were compared (Fig 2B and C). At early time points in the time window in the control embryos, sister centrosomes slid along the nuclear surface to position themselves at opposite poles of the nucleus (Fig 2C black, and Video 3), as previously reported (Gönczy et al, 1999). In the enucleated embryo, the sister centrosomes separated at a speed similar to that in the control embryos, indicating that the spacing was independent of the nucleus (Fig 2C red and Video 4). The nucleus-independent spacing was consistent with previous observations for *zyg-12* (RNAi), in which the centrosomes were not associated with the nuclei (Malone et al, 2003) (Fig 2C blue and Video 5). At later time points, for enucleated embryos, unlike the control embryos, the separation of sister centrosomes did not pause at the distance of the nuclear diameter (∼10 μm), but continued to increase. This behavior can be explained by the loss of association with the nucleus. In *zyg-12* (RNAi) embryos, the separation of the centrosomes slowed as the centrosomes formed a mitotic spindle, until the centrosomes separated again during anaphase. In conclusion, centrosomes have an intrinsic ability to separate from their sister centrosomes, independent of their sliding activity along the nuclear surface. In control embryos, the nucleus tethered the sister centrosomes. Therefore, the centrosomes do not separate further until nuclear envelope breakdown (NEBD).

Video 3 Centrosome movement in control *C. elegans* embryos during two-cell stage. Time-lapse movie corresponding to Fig 2B (control). The two-cell stage is presented. In the first five frames, yellow arrows indicate representative sister centrosomes. Time 0 was defined as the time at which representative sister centrosomes were detected. Otherwise, the imaging conditions were the same as those used in Video 1. Download video

**Figure 3.**
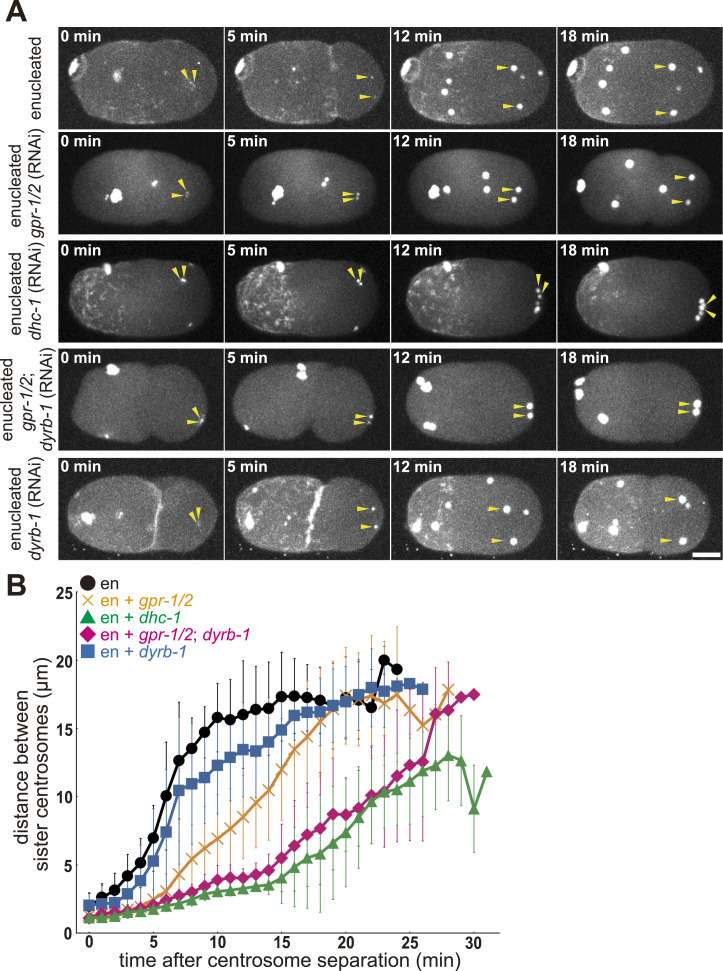
Centrosome spacing activity depends on cortical and cytoplasmic pulling forces. **(A)** Time-lapse imaging series of an embryo of an enucleated embryo, and *gpr-1/2* (RNAi)-, *dhc-1* (RNAi)-, *gpr-1/2*; *dyrb-1* (RNAi)-, and *dyrb-1* (RNAi)-enucleated embryos. The yellow arrowheads indicate a pair of sister centrosomes. z-maximum projections. The time zero is when two discrete centrosome (γ-tubulin) spots were detected for a sister pair of interest after the second centrosome duplication (described in Fig 2A). Scale bar, 10 μm. **(B)** The quantification of the distance between sister centrosomes. The mean and SD are shown with the symbol and the error bar, respectively. Black circle, enucleated embryos (10 sister pairs from 5 embryos). Orange cross, *gpr-1/2* (RNAi)-enucleated embryos (13 sister pairs from 5 embryos). Green triangle, *dhc-1* (RNAi)-enucleated embryos (12 sister pairs from 5 embryos). Magenta diamond, *gpr-1/2*; *dyrb-1* (RNAi)-enucleated embryos (12 sister pairs from 5 embryos). Blue square, *dyrb-1* (RNAi)-enucleated embryos (11 sister pairs from 5 embryos).

Video 4 Centrosome movement in enucleated *C. elegans* embryos during the two-cell stage. Time-lapse movie corresponding to Figs 2B and 3A (enucleated embryo). The imaging conditions were the same as those used for Video 3. Download video

Video 5 Centrosome movement in *zyg-12* (RNAi) *C. elegans* embryos during the two-cell stage. Time-lapse movie corresponding to Fig 2B (*zyg-12* [RNAi]). The imaging conditions were the same as those used for Video 3. Download video

**Figure S2.**
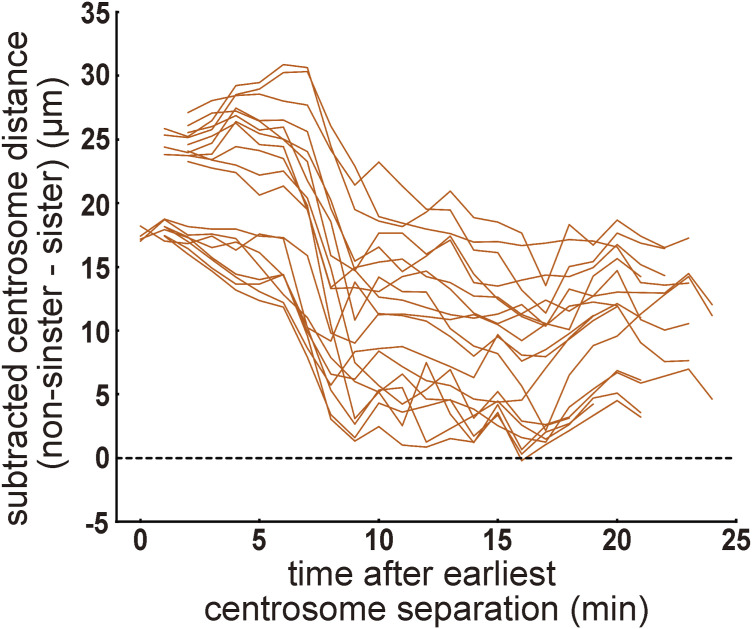
Characterization of centrosome dynamics during the second cell cycle in enucleated embryos. Related to Fig 2. Distance transition between non-sister centrosomes. The minimum sister centrosome distance was subtracted from the distance between non-sister pairs of centrosomes at each time point in the enucleated embryo. Individual samples are indicated by red lines (20 pairs of 5 embryos). The black dotted line indicates the subtracted distance of 0. The distances between non-sister pairs were rarely shorter than those between sister pairs, indicating that a similar spacing mechanism was applied to both sister and non-sister centrosome pairs.

An advantage of enucleated embryos is that the interactions between non-sister centrosomes that share the cytoplasm can be characterized. Notably, the distance between non-sister centrosomes was always longer than that between sister centrosomes (Figs 2D and S2). Although the centrosomes moved dynamically within the embryo, the distances between non-sisters did not become shorter than the minimal distance between sister pairs at each time point. The results indicated that similar spacing activity existed between sister and non-sister pairs of centrosomes. Therefore, repulsive spacing activity is intrinsic to the centrosome.

### Dynein-dependent pulling forces were responsible for the timely spacing activity

To obtain insights into the mechanism of centrosome spacing, we searched for the genes involved in this activity in enucleated embryos. Cortical pulling forces that pull microtubules from force generators located in the cell cortex contribute to centrosome separation in *Drosophila* (Cytrynbaum et al, 2003). In *C. elegans*, the knockdown of genes required to generate the cortical pulling force (e.g., *gpr-1/2* [RNAi]) impairs spacing in *zyg-12* knockdown embryos (De Simone et al, 2016). We knocked down *gpr-1/2* in an enucleated embryo to inhibit the cortical pulling force and found that the distance between the centrosomes was shortened (Fig 3A and B orange, and Video 6). A significant difference (*P* < 0.01 10-min, Wilcoxon rank-sum test) was observed between enucleated embryos and enucleated embryos with *gpr-1/2* (RNAi) in the distance between sister centrosomes. The distances for enucleated embryos and enucleated embryos with *gpr-1/2* (RNAi) were 15.8 ± 2.5 and 6.9 ± 3.2 μm (mean ± SD), n = 10 and 13, respectively, at the 10-min timing when the distance between centrosomes in enucleated embryos reached near saturation. These results indicated that the cortical pulling force mediated the spacing activity. Centrosomes were partially separated from the enucleated *gpr-1/2* (RNAi) embryos. This result is consistent with the partial separation in *zyg-12*; *goa-1/gpa-16* (RNAi) embryos (De Simone et al, 2016). It is unlikely that the remaining separation in enucleated *gpr-1/2* (RNAi) was because of the incomplete knockdown of the GPR-1/2 protein. Under our *gpr-1/2* (RNAi) conditions, P0 cells were divided equally to produce daughter cells of the same volume. This occurred when the protein level of GPR-1/2 was reduced to almost 0%, but not when the protein level was ∼30% (Pecreaux et al, 2006). These results indicated that factors other than the cortical pulling force were involved in centrosome spacing. The following observations support requirements other than the cortical pulling force.

Video 6 Centrosome movement in *gpr-1/2* (RNAi) in enucleated *C. elegans* embryos during the two-cell stage. Time-lapse movie corresponding to Fig 3A (*gpr-1/2* (RNAi)-enucleated embryo). For this individual, we did not detect the signal of *GFP*::*PH*^*PLC1δ1*^. Otherwise, the imaging conditions were the same as those used in Video 3. Download video

When *dhc-1* was knocked down in the enucleated embryo, spacing was almost completely blocked for ∼20 min, corresponding to the duration of the cell cycle in the control embryo (Fig 3A and B, green, and Video 7). *dhc-1* encodes the heavy-chain subunit of cytoplasmic dynein and is responsible for all microtubule-pulling forces in *C. elegans* embryos (Gönczy et al, 1999; Torisawa & Kimura, 2020). A significant difference was found between enucleated embryos treated with *gpr-1/2* (RNAi) and those treated with *dhc-1* (RNAi) (*P* < 0.01 10-min, Wilcoxon rank-sum test). The distance for enucleated embryos with *dhc-1* (RNAi) was 3.1 ± 0.4 μm, n = 12. Here, we focused on a near-complete block of spacing for the first 20 min under *dhc-1* (RNAi) conditions. Notably, we observed an apparent centrosome movement after 20 min. The mechanism of the latter movement is investigated in the final section of this study. Dynein inhibition impaired timely spacing activity, which occurred almost completely within 20 min. Therefore, these results suggest that factors other than the cortical pulling force, which are dependent on dynein, contribute to the spacing.

Video 7 Centrosome movement in *dhc-1* (RNAi) in enucleated *C. elegans* embryos during two-cell stage. Time-lapse movie corresponding to Fig 3A (*dhc-1* (RNAi)-enucleated embryo). The imaging conditions were the same as those used for Video 3. Download video

The cytoplasmic pulling force depends on *dhc-1* but not on *gpr-1/2*, and drives the centration of centrosomes and pronuclei (Kimura & Onami, 2005, 2007; Kimura & Kimura, 2011). We expected the cytoplasmic pulling force to contribute to this spacing. To test this possibility, the *dyrb-1* and *gpr-1/2* genes were simultaneously knocked down in enucleated embryos. *dyrb-1* encodes a roadblock subunit of the dynein complex, which is not essential for dynein motor activity but is required for organelle transport and centration of the centrosome; therefore, it is necessary for the cytoplasmic pulling force (Kimura & Kimura, 2011). In *gpr-1/2*; *dyrb-1* (RNAi)-enucleated embryos, in which cytoplasmic- and cortical-pulling forces were impaired, centrosome spacing was severely defective, as observed in *dhc-1* (RNAi)-enucleated embryos (Fig 3A and B magenta, and Video 8). A significant difference was found between embryos enucleated with *gpr-1/2* (RNAi) and embryos enucleated with *gpr-1/2*; *dyrb-1* (RNAi) in the distance between sister centrosomes (*P* < 0.01 10-min, Wilcoxon rank-sum test). The distance for enucleated embryos with *gpr-1/2*; *dyrb-1* (RNAi) was 3.9 ± 1.1 μm, n = 12. The enucleated embryos with *dyrb-1* (RNAi) repressed the spacing compared with the control (*P* < 0.05 at 10-min, Wilcoxon rank-sum test), but not as severely as *gpr-1/2*; *dyrb-1* (RNAi) (Fig 3A and B blue, and Video 9). The distance for enucleated embryos with *dyrb-1* (RNAi) was 12.3 ± 3.5 μm, n = 11. These results showed that the cortical and cytoplasmic pulling forces were sufficient to provide spacing between the centrosomes in the initial 20 min of the two-cell stage.

Video 8 Centrosome movement in *gpr-1/2*;*dyrb-1* (RNAi) in enucleated *C. elegans* embryos during two-cell stage. Time-lapse movie corresponding to Fig 3A (*dyrb-1*; *gpr-1/2* (RNAi) enucleated embryo). For this individual, we did not detect the signal of *GFP*::*PH*^*PLC1δ1*^. Otherwise, the imaging conditions were the same as those used in Video 3. Download video

Video 9 Centrosome movement in *dyrb-1* (RNAi) in enucleated *C. elegans* embryos during two-cell stage. Time-lapse movie corresponding to Fig 3A (*dyrb-1* (RNAi)-enucleated embryo). The imaging conditions were the same as those used for Video 3. Download video

### In search for a dynein-dependent mechanism for the spacing between centrosomes

In humans, *Drosophila*, and *Xenopus*, plus-end–directed motors are involved in the centrosome spacing of the mitotic spindle (see Introduction section) and are considered to be involved in chromosome-independent spacing by acting on antiparallel microtubules emanating from the two centrosomes (Deshpande et al, 2022; de-Carvalho et al, 2022). In contrast, in this study, minus-end–directed motor dynein provided the necessary force for centrosome spacing in *C. elegans* embryos. Therefore, we aimed to determine how pulling forces mediate the repulsive interactions between centrosomes.

Analogous to the finding of the antiparallel pushing mechanism for spacing activity in *Drosophila* and *Xenopus* from the mechanisms of spindle elongation, we speculated that we could obtain clues for the spacing mechanism in *C. elegans* from the mechanisms proposed for spindle elongation in the species. Spindle elongation in *C. elegans* is dynein-dependent. Most proposed models for spindle elongation in *C. elegans* embryo assume that the distribution of microtubules differs between the two centrosomes (Grill et al, 2001; Hara & Kimura, 2009). A study by Farhadifar et al proposed a mechanism called “the stoichiometric model of cortical pulling forces,” for the spindle elongation in the *C. elegans* embryo that is independent on the distribution of microtubules (Farhadifar et al, 2020). In this model, the two centrosomes of the spindle poles compete for force generators in the cell cortex to pull. “Stoichiometric” means that one force generator can pull only the nearest centrosome. This model ensures that anterior and posterior cortexes pull only the anterior and posterior centrosomes, respectively. In the present study, we applied a stoichiometric model to explain the spacing activity of four or more centrosomes in enucleated embryos.

### Quantification of the length distribution of the microtubules in the *C. elegans* embryo

**Figure 4.**
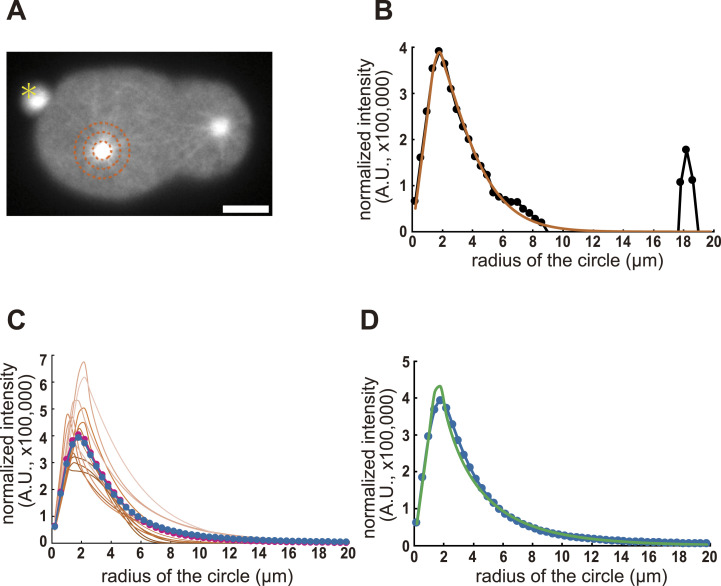
Distribution of the microtubule length in enucleated embryos. **(A)** A representative image of the β-tubulin signal in enucleated *C. elegans* embryos. The brown dot circles indicate areas 2, 4, and 6 μm from the center of the aster. The yellow asterisk indicates the polar body. Scale bar, 10 μm. **(B)** The fitting analysis result of the distribution of the β-tubulin signal in (A). The subtracted intensity value (see the Materials and Methods section for the details) is shown with the black circle and line. Brown line is the fitted curve. **(C)** The fitting results of the β-tubulin signals in an enucleated embryo. Fitted curves for each time point are shown in brown lines. Darker colors indicate earlier time points. Lighter colors indicate later time points. The average fitting curve is shown with magenta dots and lines. The average fitting curve from five embryos is shown with blue dots and lines. **(D)** The average fitting curve from five embryos is shown with blue dots and lines. The fitting of the average fitting curve is shown with a green line. The value from this result is applied for simulation (Table S2).

**Figure S3.**
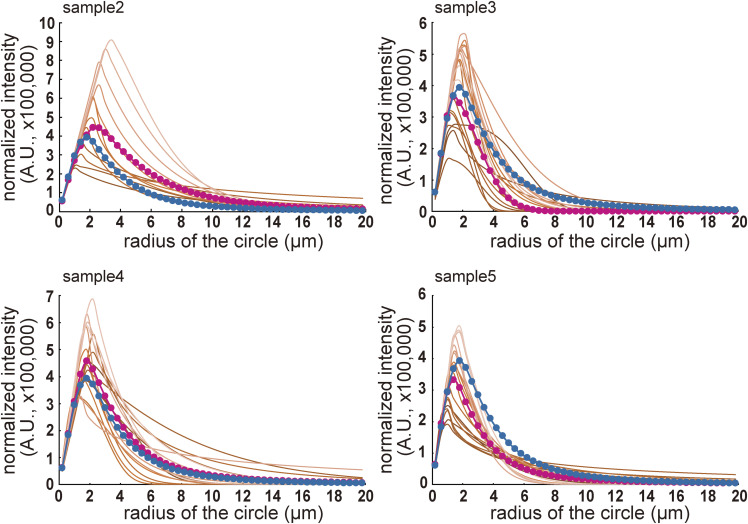
Distribution of the microtubule length in enucleated embryos. Related to Fig 4. The fitting results of the β-tubulin signals in four enucleated embryos, other than the one shown in Fig 4C (“sample 1”). The fitted curves at each time point are indicated by brown lines. Darker colors indicate earlier time points. Lighter colors indicate later time points. The average fitting curves are shown as magenta dots and lines. The average fitting curve for the five embryos is indicated by blue dots and lines. We concluded that the estimated distribution of microtubule lengths did not change dramatically among different samples.

The original stoichiometric model (Farhadifar et al, 2020) assumed long and stable microtubules (i.e., exponential decay with a characteristic length of 20 μm). The length distribution of the microtubules is critical for stoichiometric models; thus, we experimentally quantified the length distribution of the microtubules (Figs 4 and S3). We assumed that the brightness intensity of β-tubulin::GFP signal above its cytoplasmic average was proportional to the number of microtubules and quantified the value (Fig 4A and B). The quantified signal intensity fitted well with a Weibull distribution of *S*(*l*) = *S*_0_ × EXP[−{(*l* − *l*_0_)/*ξ*}^*P*], where *S*(*l*) is the signal intensity of microtubules with their length over *l*, *l*_0_ is the radius of the centrosome, *S*_0_ is the intensity at the surface of the centrosome, *ξ* is the length scale, and *P* is a parameter for how the distribution is affected by the length (Fig 4B and see the Materials and Methods section). The estimated distribution of microtubule lengths did not change dramatically during the observation period (Figs 4C and S3) or among different samples (Fig S3). Therefore, we calculated the average distribution of all samples at all the time points (Figs 4C and D and S3). Our fitting of the average distribution to the Weibull distribution revealed *l*_0_ = 1.6 μm, *ξ* = 2.3 μm, and *P* = 0.79.

### Stoichiometric model of cortical and cytoplasmic pulling forces as a mechanism for the repulsive spacing between centrosomes in the *C. elegans* embryo

**Figure 5.**
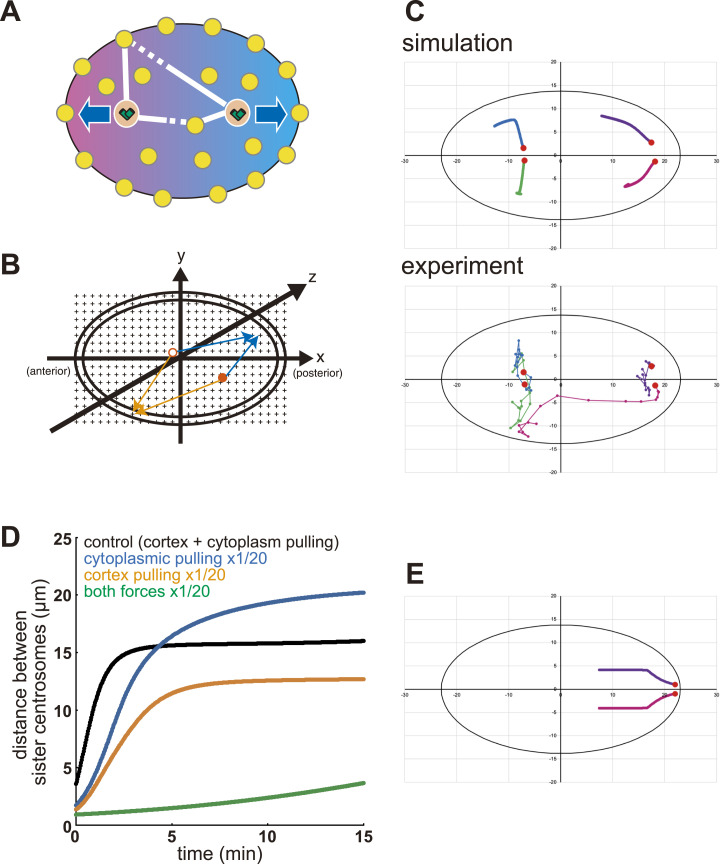
The stoichiometric model of cortical and cytoplasmic pulling forces. **(A)** Schematic of the model. Each centrosome (orange circle with two cylinders) is pulled by force generators (yellow circles) at the cell cortex and the cytoplasm. One force generator can pull only the nearest centrosome. The centrosome connected by solid lines is pulled by the force generator. **(B)** Schematic of the simulation setup. The ellipsoids represent the cell (the outer layer: the cortex, the inner mass: the cytoplasm). Red circles (open and filled) are the centrosomes. Black crosses on the lattice are the force generators evenly distributed. The force generators at the cortex, or the cytoplasm, pull the centrosomes depending on the distance between the force generator and each centrosome (orange or blue arrows, respectively). **(C)** Trajectories of the centrosomes in a representative enucleated embryo (lower) and the simulation with the same initial positions of the four centrosomes (upper). The initial positions of the centrosomes are shown with red circles. The trajectories of the same color indicate the same initial positions. **(D)** Simulated distance between the sister centrosomes in the simulation shown in (C) (black line), and in simulations with reduced (5%) cortical pulling forces (orange line), with reduced (5%) cytoplasmic pulling forces (blue line), and with a condition where both pulling forces are reduced (5% for each) (green line). **(E)** Simulation for the separation and migration of the centrosomes in the pronuclear migration stage in the WT (intact nucleus). The trajectories of the two centrosomes are shown in magenta and purple lines. The initial positions of the two centrosomes are set near the posterior end of the embryo, and the initial spacing between the centrosomes is 2 μm. The intact nucleus was simulated by restricting the distance between the two centrosomes not exceeding the nuclear diameter (8 μm).

Our analyses revealed that both cortical and cytoplasmic pulling forces act on the spacing between centrosomes (Fig 3). Therefore, we added cytoplasmic pulling forces (Fig 5A and B, and Table S2) to the stoichiometric model of the cortical pulling force by Farhadifar et al (2020). This modified version of the stoichiometric model of the cortical and cytoplasmic pulling forces reproduced the major features of our experimental measurements (Fig 5C and D). In the three-dimensional space (ellipsoid), we uniformly placed force generators in the cytoplasm and a thin layer of the cortex (Fig 5B), similar to our previous simulation (Kondo & Kimura, 2019). The centrosomes were positioned at their initial positions in representative experiments (Table S3). The simulation was conducted by iterating the microtubule growth processes, summing the pulling forces calculated as in the original stoichiometric model (Farhadifar et al, 2020) but adding the contributions from the cytoplasmic force generators, and moving the centrosomes.

Table S2 Parameters for the simulations.

Table S3 The condition-dependent parameters of the simulations.

**Figure S4.**
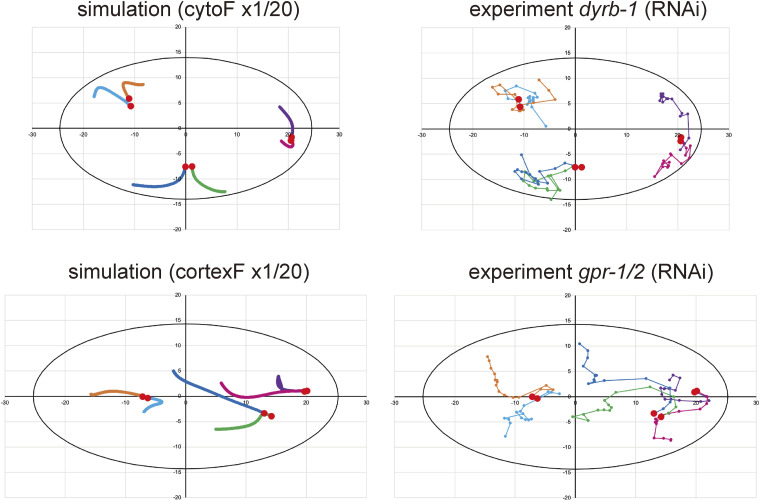
Trajectories of the centrosomes in the simulation and experiment. Related to Fig 5. Trajectories of centrosomes in a representative enucleated embryo (right) and a simulation with the same initial positions as the six centrosomes (left). The red circles indicate the initial centrosome positions. Trajectories of the same color indicate the same initial positions. The trajectories of the centrosomes inside the cells were similar in both the simulations and experiments.

For the simulation parameters, we followed the original stoichiometric model for cortical pulling forces (Farhadifar et al, 2020). See the Materials and Methods section and Table S2 for details on the parameter values. The simulations (Fig 5D) reproduced centrosome spacing of similar magnitudes and increased rates for the control, *dyrb-1* (RNAi)-, *gpr-1/2* (RNAi)-, and *dhc-1* (RNAi)-enucleated embryos, as shown in Fig 3B. The trajectories of the centrosomes inside the cells were similar between the simulations and experiments (Figs 5C and S4). The trajectories fluctuated more during the experiments, possibly because of random fluctuations in the cytoplasm (Guo et al, 2014). Overall, the numerical simulation results supported the feasibility of the stoichiometric model of the cortical and cytoplasmic pulling forces.

In addition, the stoichiometric model of the cortical and cytoplasmic pulling forces reproduced the separation and centration of the centrosomes (Fig 5E) associated with the sperm-derived pronucleus of the one-cell stage embryo (Albertson, 1984; Gönczy et al, 1999). For the simulation with the nucleus, the two centrosomes were connected with an elastic bar equal to the length of the nuclear diameter (8 μm). This setup allows the centrosomes to attract each other when the distance between them exceeds the nuclear diameter. This result supports the feasibility of the model even for cells with nuclei.

**Figure S5.**
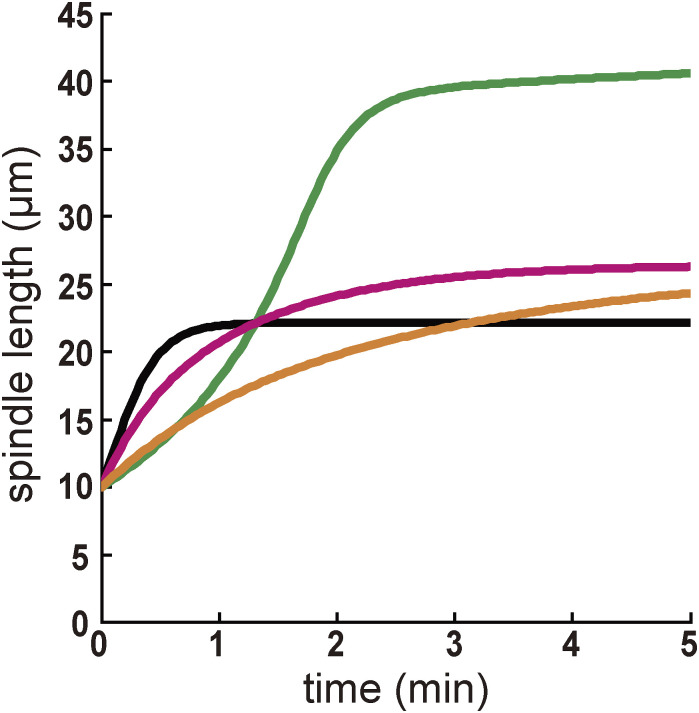
Stoichiometric model of cortical and cytoplasmic pulling forces reproduces spindle elongation. Related to Fig 5. Simulated results for the spindle length. The line indicates simulated spindle length. Black: simulation only with cortical pulling forces (*f*_*0_cort*_ = 0.08), without cytoplasmic pulling forces (*f*_*0_cyto*_ = 0), and with long microtubules (exponential decay with a characteristic length of 20 μm) as assumed in Farhadifar et al (2020). Green: simulation as in black, except for the experimentally obtained microtubule distribution (Fig 4D and Table S2). Magenta: simulation as in green, except for the cytoplasmic pulling forces (*f*_*0_cyto*_ = 0.033). Orange: simulation as in magenta, except using the force parameters as in our other simulations (*f*_*0_cort*_ = 0.034 and *f*_*0_cyto*_ = 0.014, Figs 5 and S4, and Table S2).

Application of the experimentally observed length distribution of the microtubules (Fig 4) to the original stoichiometric model resulted in the elongation of the spindle for almost as long as the cell length (Fig S5). This is likely because the microtubules in the cell are shorter than the distribution assumed in the original simulation by Farhadifar et al (2020). The addition of cytoplasmic pulling forces to the original stoichiometric model enabled reasonable elongation with the microtubule lengths consistent with the experiment (Fig S5), indicating that the stoichiometric model of cortical and cytoplasmic pulling forces accounts for spindle elongation, in addition to the separation and centration of centrosomes in normal embryos with nuclei and chromosomes.

### Myosin-dependent movements of the centrosomes in the *C. elegans* embryo

**Figure 6.**
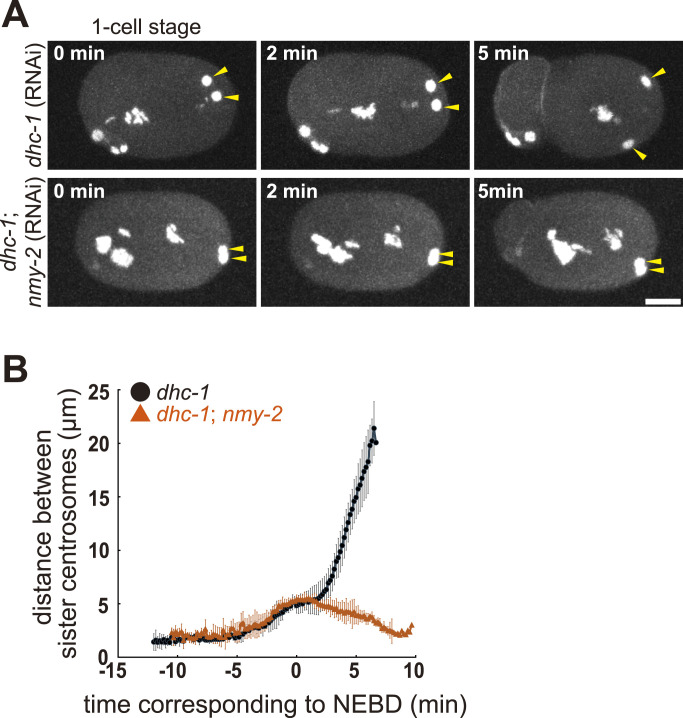
The myosin-dependent movement of the centrosomes. **(A)** Time-lapse imaging series of a *dhc-1* (RNAi) embryo, and a *dhc-1*; *nmy-2* (RNAi) embryo of the DE90 strain at one-cell stage. GFP-labeled γ-tubulin (centrosome), histone H2B (chromosome), and PH^PLCδ1^ (cell membrane) are shown. The yellow arrowheads indicate a pair of sister centrosomes. z-maximum projections. Scale bar, 10 μm. **(B)** The quantification of the distance between sister centrosomes. The mean and SD are shown by the symbol and error bar, respectively. Black circle, *dhc-1* (RNAi) embryos (6 sister pairs from 6 embryos). Red triangle, *dhc-1*; *nmy-2* (RNAi) embryos (5 sister pairs from 5 embryos).

In this study, large movement of centrosomes was observed ∼20 min after the detection of the two centrosomes in *dhc-1* (RNAi)-enucleated embryos (Fig 3B green, Video 7). In *dhc-1* (RNAi) embryos with nuclei, the centrosomes did not separate during interphase at the one-cell stage, and a small spindle-like structure was formed near the cortex, suggesting that dynein was responsible for all centrosome movements until the spindle formation stage in normal embryos (Fig 6A). We noticed that the centrosomes moved over a large distance in *dhc-1* (RNAi) embryos with nuclei in the later cell cycle phase, indicating that large centrosome movement independent of *dhc-1* was not specific to enucleated embryos (Video 7 and Video 10 [left]).

Video 10 Centrosome movement in *dhc-1* (RNAi) and *dhc-1*;*nmy-2* (RNAi) *C. elegans* embryos during one-cell stage. Time-lapse movie corresponding to Fig 5A (left, *dhc-1* (RNAi) embryo; right, *nmy-2*; *dhc-1* (RNAi) embryo). The *C. elegans* embryo contains the nuclei and expressing *GFP*::*histone H2B*, *tbg-1*::*GFP*, *GFP*::*PH* ^*hPLCIIIδ1*^. In the first 10 frames, the yellow arrows indicate representative sister centrosomes. One-cell stage imaging video is shown. z-maximum projections. Time 0 was when the imaging started. The time interval between measurements was 10 s. Scale bar, 10 μm. Download video

A large movement occurred near the time of cytokinesis in *dhc-1* (RNAi) embryos; therefore, we speculated the involvement of actomyosin, which drives the constriction of the contractile ring and the accompanying cytoplasmic flow toward the equatorial plane (Bray & White, 1988; Khaliullin et al, 2018). Although cytokinesis does not occur at this stage in enucleated embryos, the cytoplasmic flow may move the centrosomes. Unfortunately, an enucleated embryo cannot be obtained upon knockdown of *nmy-2*, which encodes non-muscle myosin II and is required for cytoplasmic flow (Munro et al, 2004). This was possibly because NMY-2 is required for polar body extrusion (Dorn et al, 2010) and *nmy-2* (RNAi) impairs oocyte enucleation. To this end, *dhc-1* and *nmy-2* were simultaneously knocked down in the nucleated embryos. We observed an impairment in dynein-independent centrosome movements, indicating that large movements were driven by cytoplasmic flow (Fig 6A and B, and Video 10 [right]). Differences in the distance between centrosomes 5 min after NEBD were tested using the Wilcoxon rank-sum test between *dhc-1* (RNAi) and *dhc-1*; *nmy-2* (RNAi) experiments (*P* < 0.01). The mean values for *dhc-1* (RNAi) and *dhc-1*; *nmy-2* (RNAi) were 14.9 ± 2.0 and 3.9 ± 0.9 μm, n = 6 and 5, respectively. In conclusion, cytoplasmic flow drives centrosome movement during late mitosis, which occurs when dynein is inhibited.

## Discussion

### Enucleation of *C. elegans* embryos

Chromosomes are essential for cellular functions because they carry genetic information and constitute a core component of intracellular organelles. Before NEBD, chromosomes form a cell nucleus, whereas after NEBD, they form a mitotic spindle. Historically, chromosome removal from cells has enabled important modeling in cell biology studies (Goldman & Pollack, 1974). In addition to the mechanical removal of the nucleus (e.g., by centrifugation or microneedles), genetic manipulation can also be used to investigate the function of nuclei. The *gnu* mutant of *Drosophila* revealed a semi-enucleated system in *Drosophila* embryos, in which the nucleus did not divide but formed one giant nucleus, whereas the centrosomes were continually duplicated and separated (Freeman et al, 1986). In *gnu* mutants, centrosome separation was almost entirely independent of the presence of nuclei (de-Carvalho et al, 2022). In the present study, we established a genetic method to obtain enucleated *C. elegans* embryos by combining previously established methods to remove chromosomes from the sperm (Sadler & Shakes, 2000) and oocytes (Segbert et al, 2003) (Fig S1). Our established method produces enucleated embryos in a noninvasive manner, whereas classical enucleated *C. elegans* embryos must be obtained by penetrating the eggshell using laser microsurgery (Schierenberg & Wood, 1985). Unlike the *Drosophila gnu* mutant, our method completely removed chromosomes from embryos. We expect our established method to be applied in various studies and not limited to centrosome research because *C. elegans* embryos are widely used model organisms.

### Chromosome-independent and dynein-dependent spacing between the centrosomes

Using an enucleated *C. elegans* embryo, we demonstrated that spacing activity, independent of the chromosome, exists in the *C. elegans* embryo before and after NEBD and between sister and non-sister centrosomes. Nucleus-independent spacing between sister centrosomes before NEBD has been previously observed in the *zyg-12* mutant, in which the association between the nucleus and centrosomes is impaired (Malone et al, 2003; De Simone et al, 2016). In contrast, as the mitotic spindle forms and most cells divide in *zyg-12* mutants, the chromosome-independent interaction after NEBD and that for non-sister centrosomes have not been previously addressed. In addition, even in *zyg-12* mutants, nuclei remain in the cytoplasm between the centrosomes during interphase, which acts as an obstacle to microtubule growth in these regions. Therefore, the enucleated embryo is a good model system for studying the interactions between centrosomes in an intracellular space without physical obstacles.

We demonstrated that the spacing between sister and non-sister centrosomes until cytokinesis completely impaired the knockdown of dynein (*dhc-1*). This result indicated that kinesin-dependent pushing between centrosomes, as revealed in *Drosophila* and *Xenopus* (Telley et al, 2012; Nguyen et al, 2014, 2018), did not occur in *C. elegans* embryo. Our observations in *C. elegans* is consistent with previous research showing that centrosomes rarely moved in *dhc-1* (RNAi) embryos (Gönczy et al, 1999) and that the molecules involved in pushing did not impair the elongation of the mitotic spindle in *C. elegans* embryo (Powers et al, 2004; Saunders et al, 2007; Lee et al, 2015). Our study using enucleated embryos demonstrates the direct requirement of dynein (*dhc-1*) for centrosome spacing.

### Stoichiometric model of cortical and cytoplasmic pulling forces

The major involvement of dynein indicates that the centrosome spacing in *C. elegans* is driven by a pulling force outside the centrosome pairs. The separation of centrosomes by outward pulling forces commonly occurs before NEBD (Gönczy et al, 1999; Cytrynbaum et al, 2003; De Simone et al, 2016) or spindle elongation (anaphase B) (Grill et al, 2001). However, it has not yet been determined why the two adjacent centrosomes are pulled in opposite directions. In mitotic spindles, the spindle itself exhibits bipolarity, and this difference is established during spindle formation. In other cases, the cell nucleus may amplify the asymmetry by positioning itself between the centrosomes and obstructing the growth of microtubules toward the nucleus (Donoughe et al, 2022). Cortical flow has also been proposed to separate the centrosomes (De Simone et al, 2016); however, the mechanism that ensures centrosome movement in the opposite direction has not yet been clarified.

Here, we extend the idea of the stoichiometric model of cortical pulling forces proposed by Farhadifar et al (2020) for spindle elongation to explain the spacing activity independent of nuclei and spindles. An important modification was the addition of a cytoplasmic pulling force. The importance of the cytoplasmic pulling force was demonstrated in this study using two independent supports. First, DYRB-1, a molecule involved in the generation of the cytoplasmic pulling force, was required for centrosome separation after knocking down the cortical pulling force via *gpr-1/2* (RNAi) (Fig 3). The numerical models with the cytoplasmic pulling force, but not the models with only the cortical pulling force, accounted for the centrosome spacing, and the centrosome separation and spindle elongation, with the experimentally obtained distribution of microtubule length (Figs 4 and S5). The cytoplasmic pulling force contributes to the centrosome spacing even when the microtubules are short and not reaching the cortex. Therefore, the cytoplasmic pulling force may be important for the rapid placement of centrosomes during development, and for cell types with asters that are formed only of short microtubules.

A stoichiometric model is proposed as the underlying mechanism for spindle elongation (Farhadifar et al, 2020). The idea that a force generator can pull only one microtubule among multiple microtubules, potentially reaching the force generator, is in line with a previously proposed force-generator–limited model (Grill et al, 2003; Grill & Hyman, 2005). We noticed that during spindle elongation, the microtubules from the two spindle poles rarely overlap. This observation indicated that the competition assumed in the stoichiometric model may not be critical for spindle elongation. In contrast, in enucleated embryos, there was no apparent bias in the direction of microtubule elongation. We demonstrate that a stoichiometric model of cortical and cytoplasmic pulling forces is critical for centrosome spacing in *C. elegans* embryos. Because we demonstrated the existence of spacing activity and a stoichiometric model of cortical and cytoplasmic pulling forces as the underlying mechanisms, the model ensured spindle elongation even without the formation of mitotic spindles (Fig S5).

The stoichiometric model of cortical and cytoplasmic pulling forces corroborated the spacing dynamics of centrosome pairs in control and gene knockdown-enucleated embryos (Figs 5 and S4). Moreover, the model explained the separation and centering of the normal embryo with the nucleus when the centrosomes were tethered to the nuclear surface (Fig 5E). Therefore, the stoichiometric model of cortical and cytoplasmic pulling forces is promising for centrosome spacing in *C. elegans* embryos and can be applied to other cell types and species.

A similar pulling force-based mechanism has been proposed for the spacing of nuclei in the syncytium embryo of the cricket *Gryllus bimaculatus* (Donoughe et al, 2022), despite the observation of a pushing-based mechanism for similar nuclear spacing in *Drosophila* syncytium embryos (Deshpande et al, 2022). The proposed pulling-based mechanism in crickets supports the generalizability of the mechanism proposed in this study to *C. elegans* embryos. However, further studies are necessary to clarify pulling-based mechanisms in crickets. The involvement of dynein and other pulling force generators in crickets has not yet been demonstrated. The current argument against the pushing-based mechanism in crickets is that numerical simulations (Dutta et al, 2019) do not reflect certain aspects of nuclear migration (Donoughe et al, 2022). It is possible that kinesin-5, PRC1, or kinesin-4 is required for spacing in crickets. The pulling-based model proposed for crickets (Donoughe et al, 2022) is similar to that used in the present study. Unlike our model, which is independent of the nucleus, the model for the cricket assumed occlusion of the microtubule “cloud” by the nucleus as the primary driving force. In *Drosophila*, centrosome spacing in the syncytium is independent of the nucleus (de-Carvalho et al, 2022), which may also be the case in crickets. In this scenario, a stoichiometric model of the cortical and cytoplasmic pulling forces that does not require nuclei for centrosome spacing would be more suitable, even for crickets. Finally, in contrast to the cricket model, in which an occlusion between the microtubule “clouds” was assumed without mechanistic bases, the stoichiometric model of cortical and cytoplasmic pulling forces assumes competition based on the reasonable length distribution of the microtubule (i.e., longer microtubules are rare). In this regard, we believe that the stoichiometric model of the cortical and cytoplasmic pulling forces is more widely applicable. The pulling-based mechanism may be used in combination with repulsive pushing between centrosomes using antiparallel microtubules in other cell types and organisms.

### Myosin-dependent centrosome movement

We observed a large movement of centrosomes in dynein (*dhc-1*)-knockdown embryos (Fig 6). Previous studies have focused on some of the earliest phenotypes (defects in centrosome separation, pronuclear migration, and spindle elongation in the one-cell stage embryo) of *dhc-1* RNAi or mutant embryos (Gönczy et al, 1999; Kimura & Onami, 2005; Schmidt et al, 2005), but have not focused on the later movements of the centrosomes. The timing of the large centrosome movement in *dhc-1* (RNAi) embryos coincides with that of cytokinesis. This suggests the involvement of cytoplasmic flow coupled with cytokinesis (White & Borisy, 1983; Khaliullin et al, 2018). This idea is supported by our RNAi experiment on non-muscle myosin *nmy-2*, a gene responsible for generating cytoplasmic flow (Shelton et al, 1999). We confirmed that cytoplasmic flow occurred in *dhc-1* (RNAi) cells.

The involvement of the cytoplasmic flow in centrosome movement (with or without nuclei) has been previously reported. In the one-cell stage *C. elegans* embryo, soon after symmetry breaking, the centrosomes moved along the cortex in *zyg-12* (RNAi) embryos in an *nmy-2*-dependent manner (De Simone et al, 2016). This dependence on *nmy-2* suggests that cytoplasmic flow is the driving force for this movement. However, an alternative interpretation is possible. Because *nmy-2* (RNAi) impaired cortical pulling forces (Redemann et al, 2010), and defective cortical pulling forces impaired movement along the cortex (Figs 5C and S4, compare control-versus-*gpr-1/2* [RNAi]), the centrosome movement behavior in *zyg-12*; *nmy-2* (RNAi) (De Simone et al, 2016) can be explained by defects in the cortical pulling force.

Another example of centrosome movement by cytoplasmic flow is from the one-cell stage *C. elegans* embryo, but occurs earlier than symmetry breaking. Cytoplasmic flow, driven by kinesin and microtubules (McNally et al, 2010; Kimura et al, 2017), moves the sperm-derived pronucleus together with the centrosome, affecting the formation of the anterior–posterior axis of the embryo (Kimura & Kimura, 2020). In *Drosophila* syncytium embryos, nuclear movement via myosin-dependent cytoplasmic flow is important for nuclear positioning and synchronized cell division (von Dassow & Schubiger, 1994; Deneke et al, 2019). Our findings of centrosome movement by cytoplasmic flow may provide insights into how cytoplasmic flow moves the nucleus and centrosomes in future studies.

## Materials and Methods

### Worm strains and RNAi

The *C. elegans* strains used in this study are listed in Table S1. The DE90 strain (*tbg-1*::*GFP*; *GFP*::*histone H2B*; *GFP*::*PH*^*PLCδ1*^) was used to obtain embryos with nuclei (*zyg-12*, *dhc-1* and *dhc-1*; *nmy-2* RNAi experiments). The strains were maintained under standard conditions (Brenner, 1974). Knockdown of *klp-18*, *zyg-12*, *gpr-1/2*, *dhc-1*, *dyrb-1*, and *nmy-2* was performed using the injection RNAi method, as previously described (Kimura & Kimura, 2011). For double- or triple-RNAi experiments, RNA was mixed at a 1:1 or 1:1:1 ratio and injected into the worms. The dsRNA concentrations were 18 or 21 μg/μl for *klp-18*, 15 or 19 μg/μl for *gpr-1/2*, 19 μg/μl for *dhc-1*, and 13 μg/μl for *dyrb-1*. To efficiently obtain the *klp-18* phenotype (enucleated embryos), observations were started ≧24 h after injection. Observations were also conducted at ≧26 h after double knockdown and ≧30 h after triple knockdown. The worms were incubated at 25°C for ≧16 h before observation (*zyg-12*, *dhc-1* and *dhc-1*; *nmy-2* RNAi experiments).

### Production of enucleated embryos

The enucleated embryos were produced as follows (Fig S1). First, two young adults each of the CAL0051, CAL0181, and CAL2741 strains were transferred to a new plate 5 d before day 1 and preculture was initiated. On day 1, 15 CAL0051 and 10 CAL0181 or CAL2741 young adults were separately transferred onto new 6-cm (diameter) NGM plates with the OP50 *E. coli*, and the plates were cultured at 16°C, a nonrestrictive temperature. After 24 h, on day 2, the adult worms were removed from the plate, and only embryos that had been laid in the last 24 h remained on the plate. Cultures were maintained at 16°C. On day 3, 24 h after the procedures on day 2, the plates were moved to 25°C, which is the restrictive temperature. On day 4 and 24 h after the procedures on day 3, 25 CAL0181 or CAL2741 L4, or young adults were selected (hermaphrodites with vulva) and injected with *klp-18* dsRNA. After injection, culturing on the NGM plate was continued with five times the number of CAL0051 males added (25 hermaphrodites and 125 males). A 3.5-cm NGM plate was used for mating. Finally, on day 5 and 24 h or more after injection, the worms were dissected, and the embryos were observed under a confocal microscope.

### Microscopic observation

The localization of fluorescent proteins was observed using a spinning-disk confocal microscope (CSU-MP; Yokogawa Electric) (Otomo et al, 2015; Kamada et al, 2022) equipped with an EM-CCD camera (iXon; Andor) mounted on an IX71 microscope (Olympus) and controlled using NIS-elements software (Nikon). Details of the system and the examination of phototoxicity will be published elsewhere (in preparation). Dissected worm embryos were attached to a poly L-lysine–coated cover glass, mounted under a microscope, and observed using a 40× objective lens at 2× intermediate magnification.

To analyze the centrosome distance, two-photon excitation with a 920-nm laser (ALCOR920-2; Spark Lasers) was used with 96-ms exposure. For Figs 1–3, and related supplemental figures and movies, 61 or 71 images were taken at 0.5-μm intervals on the z-axis. Time-lapse images were collected at 1-min intervals for less than 2 h. For Fig 6, 41 images were taken at 0.5-μm intervals along the z-axis. Time-lapse images were collected at 10-s intervals during the one-cell stage.

To analyze the distribution of microtubules and centrosomes (Figs 4 and S3), dissected worm embryos were attached to a 2% agarose-coated cover glass and mounted on a microscope. A single focal plane was captured using single-photon excitation with a 488 nm laser with 804-ms exposure. The focal plane was manually adjusted to the target aster during this interval. Time-lapse images were collected at ∼1-min interval for 30 min.

Under these microscopic conditions, *C. elegans* embryos were confirmed to hatch. Captured images were analyzed using ImageJ/Fjii or Imaris software (Oxford Instruments).

### Measurement of centrosome distance

The distance between the centrosomes was quantified using Imaris 3D analysis software. Centrosome signals were manually tracked using the spot-tracking mode. The centroid coordinates of the centrosome signals were calculated using a software. The distances between the centrosomes were calculated using the calculated coordinates. For the control, *zyg-12* (RNAi), and enucleated embryos (Figs 2C and D and 3B), the centrosomes (Fig 2A), which split into two at the two-cell stage, were tracked until the signal became undetectable or until the next duplication occurred. As shown in Fig 6, centrosome signals during the one-cell stage were tracked until they became undetectable.

### Analysis of microtubule distribution

For β-tubulin::GFP images (Fig 4A), the center coordinates of the centrosomes were quantified using the SpotTracker plugin in ImageJ (Sage et al, 2005) (http://bigwww.epfl.ch/sage/soft/spottracker/). The fluorescence intensity of soluble β-tubulin was defined as the peak intensity of the cytoplasmic signal. The fluorescence intensity of polymerized β-tubulin (i.e., microtubules) was defined as the captured intensity subtracted by the soluble β-tubulin intensity. The mean and SEM of the subtracted intensities were calculated for the ring-shaped region for every four-pixel thickness. The mean intensity was then multiplied by the average ring circumference. We assumed that the summed intensity of the ring regions, *S*(*R*), was proportional to the number of microtubules reaching the rings, and plotted *S*(*R*) against the average radius of the ring, *R* (Fig 4B). The plot was fitted with a combination of two functions: *S*(*R*) = *a* × *R* (for *R* < *R*_0_) and *S*(*R*) = *a* × *R*_0_ × EXP[−{(*R* − *R*_0_)/*ξ*}^*P*] (for *R* ≧ *R*_0_), where *a*, *R*_0_, *ξ* and *P* are the fitting parameters. Fitting was conducted using the maximum likelihood method (Yesbolatova et al, 2022), assuming that the error was normally distributed, with its mean summed-intensity and variance as the square of the SEM multiplied by the circumference length, using the solver function of Microsoft Excel (Microsoft Corporation). The function *S*(*R*) = *a* × *R*_0_ × EXP[−{(*R* − *R*_0_)/*ξ*}^*P*] represents the Weibull distribution. The Weibull distribution was used to model the distribution of the lifespan, whose death rate is proportional to the power of time. We confirmed that the simulated microtubule length distribution with constant growth/shrinkage velocity and catastrophe/rescue frequency fit well with the Weibull distribution. Therefore, we determined the length (*l*) distribution of the microtubule as *S*(*l*) = *a* × *l*_0_ × EXP[−{(*l* − *l*_0_)/*ξ*}^*P*] (for *l* ≧ *l*_0_, where *l*_0_ is the radius of the centrosome).

To calculate the average distribution of microtubule length at every time point and sample, we first fitted the microtubule length distribution at each time point for each sample using the Weibull distribution. After fitting, the average value of the fitted distribution is calculated to obtain the average distribution. This average distribution was further fitted to a Weibull distribution to obtain function parameters. The microtubule length distribution in our simulation was obtained using a Weibull distribution.

### Statistical analysis

The distances between centrosomes were statistically compared using the Wilcoxon rank-sum test, which was performed for two experimental groups of interest. Calculations were performed using the “ranksum” function of MATLAB software (The Mathworks).

### Numerical simulation of the stoichiometric model of cortical and cytoplasmic pulling forces

The settings of our previous simulation (Kondo & Kimura, 2019) were modified to model the embryo as a 3D ellipsoid with the measured lengths of the long axis and the two short axes (Table S3). As in the previous simulation, we distributed “force generation points” throughout the cytoplasm and the cortex (3-μm thick layer) at the vertices of a simple cubic lattice with 1-μm intervals. When a force generation point was associated with a microtubule elongating from the centrosome, the centrosome was pulled with a defined force (Table S2).

The probability of the point attaching a microtubule from the *i*-th centrosome was defined by the distance between the point and the centrosomes, as assumed in the stoichiometric model of cortical pulling forces proposed by Farhadifar et al (2020): $P_{i}=\frac{\Omega \left(d_{i}\right)}{\overset{N}{\underset{j}{\sum }}\Omega \left(d_{j}\right)+\kappa }$, where *Ω*(*d*_i_) is the rate microtubules from *i*-th centrosome contact a force-generator at a distance of *d*_i_, and *κ* is the rate a force generator detach from a microtubule. There are three notable differences between the original stoichiometric model (Farhadifar et al, 2020) and the stoichiometric model of the cortical and cytoplasmic pulling forces in this study. First, the model was extended to simulate the behavior of more than three centrosomes. Second, the force generators pull microtubules in the cortex and cytoplasm, based on our experimental results showing that simultaneous knockdown of *gpr-1/2* and *dyrb-1* but not either, is required for spacing defects comparable with those of *dhc-1* (RNAi) (Fig 3). Third, we used the distribution of microtubule lengths based on our own experimental measurements of signal intensity, reflecting the microtubule distance, *d*, from the center of the centrosome: *S*(*d*) = *a* × *d*_0_ × EXP[−{(*d* − *l*_0_)/*ξ*}^*P*] (for *d* ≧ *l*_0_), where *l*_0_ is the radius of the centrosome (Fig 4 and Table S2). Finally, we defined *Ω*(*d*) as *Ω*(*d*) = (*γ*/4)(*r*/*d*)^2^ × EXP[−{(*d* − *l*_0_)/*ξ*}^*P*] (for *d* ≧ *l*_0_), where *γ* is the rate of microtubule nucleation at the centrosome and *r* is the force-generator capture radius. For the case where the force generator is located inside the centrosome (*d* < *l*_0_), we assumed that all the nucleated microtubules reach the distance, and thus defined *Ω*(*d*) as *Ω*(*d*) = (*γ*/4)(*r*/*d*)^2^ (for *d* < *l*_0_).

Once we calculated the probability *P*_i_, for each force generator to pull the *i*-th centrosome, the force that pulls the *i*-th centrosome was calculated as *f*_0_*P*_i_, where *f*_0_ is the force generated by each force generator (Farhadifar et al, 2020). In this study, we defined *f*_0_cort_ and *f*_0_cyto_ as the forces generated by the cortical and cytoplasmic force generators, respectively. The total force vector acting on each centrosome was calculated by summing all the force vectors of the force generators and pulling the centrosome in the direction of each force generator. After summing the forces acting on each centrosome, the velocity of the movement was calculated as $\vec{v}=\vec{F}/\eta$, where $\vec{F}$, *η*, and $\vec{v}$ are the force, drag coefficient, and velocity vector, respectively. The positions of the centrosomes in the next step were calculated as $\vec{c_{t+\Delta t}}=\vec{c_{t}}+\vec{v}\times \Delta t$, where $\vec{c_{t}}$ and $\vec{c_{t+\Delta t}}$ are the position vectors of the centrosomes at times *t* and *t*+Δ*t*, respectively, and Δ*t* is the time interval. This calculation was repeated for the defined steps starting from the initial positions of the centrosomes.

When the centrosomes were tethered to the surface of the nucleus, we added an additional process after each step to apply an elastic force, $\vec{F_{1}}=-k_{s}\left(\vec{c_{1}}-\vec{c_{2}}\right)$ if $L>\left|\vec{c_{1}}-\vec{c_{2}}\right|$ to maintain the distance between the centrosomes at *L* or shorter. Here, $\vec{c_{1}}$ and $\vec{c_{2}}$ are the position vectors of the centrosome and other centrosomes, respectively. *k*_*s*_ is the elastic constant and *L* is the diameter of the nucleus (8 μm).

The simulation was coded using MATLAB, and the codes are available upon request.

### Parameter values of the numerical simulation

The parameter values used for the numerical simulations are summarized in Tables S2 and S3. We followed the embryo geometry based on the experimental measurements and the simulation setup in our previous study (Kondo & Kimura, 2019). The parameters for force generation were essentially identical to those used by Farhadifar et al (2020). In our setup, the number of cortical force generators (*N*_cort_) was 12,410 under the control condition. According to Farhadifar et al (2020), the pulling force produced by a force generator (*f*_0_cort_) was 0.08 pN, the force generator capture radius (*r*) was 0.1 μm, and the microtubule force generator detachment rate (*κ*) was 4.4 × 10^−4^/s.

**Figure S6.**
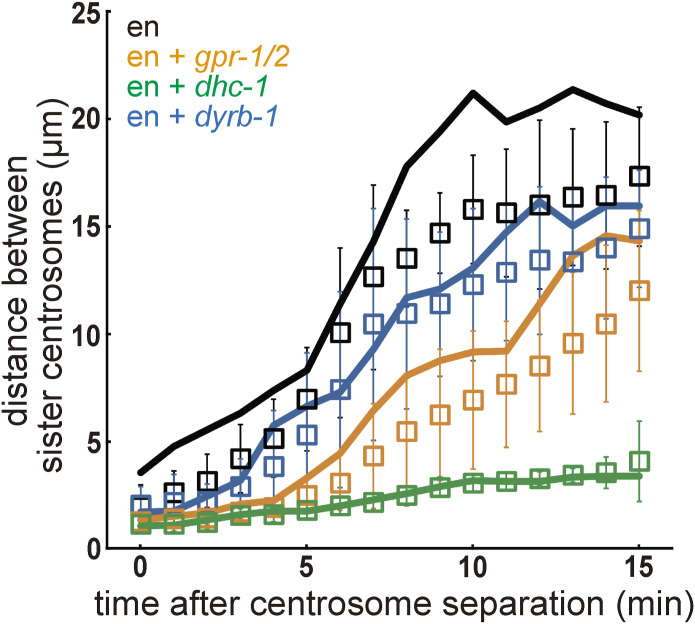
Mean distance between sister centrosomes in the representative enucleated embryos. Related to Fig 5. The mean distance between sister centrosomes in a representative embryo under each condition is indicated by a solid line. The mean and SD of all embryos, which were identical to the results shown in Fig 3B, are shown as squares and error bars, respectively. Black, enucleated embryos. Orange, *gpr-1/2* (RNAi)-enucleated embryos. Green, *dhc-1* (RNAi)-enucleated embryos. Blue, *dyrb-1* (RNAi)-enucleated embryos.

To determine the cortical pulling force, cytoplasmic pulling force, and force reduction by RNAi, we compared the simulated results for the distance between sister centrosomes under different conditions (control, reduced cortical pulling force, and reduced cytoplasmic pulling force) with the corresponding experimental results (control, *gpr-1/2*, *dyrb-1*; Figs 3B and S6). First, we searched for appropriate values of the force produced by a cortical force generator (*f*_0_cort_) and a cytoplasmic force generator (*f*_0_cyto_) that reproduced the maximum rate of increase in distance for *dyrb-1* (RNAi)-enucleated embryos (i.e., defective cytoplasmic forces) and *gpr-1/2* (RNAi)-enucleated embryos (i.e., defective cortical forces) (the number of cytoplasmic force generators (*N*_cyto_) in our setup was 18,318 under control conditions). The average values of the optimized force parameters for the three pairs of representative embryos were 0.034 pN for *f*_0_cort_ and 0.014 pN for *f*_0_cyto_. Using these parameter values, we simulated centrosome movement in the control, *dyrb-1* (RNAi)-, *gpr-1/2* (RNAi)-, and *dhc-1* (RNAi)-enucleated embryos. To mimic the low-level spacing in *dhc-1* (RNAi)-enucleated embryos, we assumed that RNAi treatment reduced the force (*f*_0_cort_ and *f*_0_cyto_) to 5%, but not to 0%.

## Supplementary Material

Reviewer comments

## References

[bib1] Albertson DG (1984) Formation of the first cleavage spindle in nematode embryos. Dev Biol 101: 61–72. 10.1016/0012-1606(84)90117-9 6692980

[bib2] Azimzadeh J, Bornens M (2007) Structure and duplication of the centrosome. J Cell Sci 120: 2139–2142. 10.1242/jcs.005231 17591686

[bib3] Baker J, Theurkauf W, Schubiger G (1993) Dynamic changes in microtubule configuration correlate with nuclear migration in the preblastoderm Drosophila embryo. J Cell Biol 122: 113–121. 10.1083/jcb.122.1.113 8314839 PMC2119602

[bib4] Bray D, White JG (1988) Cortical flow in animal cells. Science 239: 883–888. 10.1126/science.3277283 3277283

[bib5] Brenner S (1974) The genetics of Caenorhabditis elegans. Genetics 77: 71–94. 10.1093/genetics/77.1.71 4366476 PMC1213120

[bib6] Bringmann H, Hyman AA (2005) A cytokinesis furrow is positioned by two consecutive signals. Nature 436: 731–734. 10.1038/nature03823 16079852

[bib7] Brust-Mascher I, Civelekoglu-Scholey G, Kwon M, Mogilner A, Scholey JM (2004) Model for anaphase B: Role of three mitotic motors in a switch from poleward flux to spindle elongation. Proc Natl Acad Sci U S A 101: 15938–15943. 10.1073/pnas.0407044101 15522967 PMC524698

[bib8] Cheng X, Ferrell JE (2019) Spontaneous emergence of cell-like organization in Xenopus egg extracts. Science 366: 631–637. 10.1126/science.aav7793 31672897 PMC7839252

[bib9] Cytrynbaum EN, Scholey JM, Mogilner A (2003) A force balance model of early spindle pole separation in Drosophila embryos. Biophys J 84: 757–769. 10.1016/s0006-3495(03)74895-4 12547760 PMC1302656

[bib10] De Simone A, Nédélec F, Gönczy P (2016) Dynein transmits polarized actomyosin cortical flows to promote centrosome separation. Cell Rep 14: 2250–2262. 10.1016/j.celrep.2016.01.077 26923593

[bib11] de-Carvalho J, Tlili S, Hufnagel L, Saunders TE, Telley IA (2022) Aster repulsion drives short-ranged ordering in the Drosophila syncytial blastoderm. Development 149: dev199997. 10.1242/dev.199997 35001104

[bib12] Deneke VE, Puliafito A, Krueger D, Narla AV, De Simone A, Primo L, Vergassola M, De Renzis S, Di Talia S (2019) Self-organized nuclear positioning synchronizes the cell cycle in Drosophila embryos. Cell 177: 925–941.e17. 10.1016/j.cell.2019.03.007 30982601 PMC6499673

[bib13] Deshpande O, de-Carvalho J, Vieira DV, Telley IA (2022) Astral microtubule cross-linking safeguards uniform nuclear distribution in the Drosophila syncytium. J Cell Biol 221: e202007209. 10.1083/jcb.202007209 34766978 PMC8594625

[bib14] Dogterom M, Yurke B (1997) Measurement of the force-velocity relation for growing microtubules. Science 278: 856–860. 10.1126/science.278.5339.856 9346483

[bib15] Donoughe S, Hoffmann J, Nakamura T, Rycroft CH, Extavour CG (2022) Nuclear speed and cycle length co-vary with local density during syncytial blastoderm formation in a cricket. Nat Commun 13: 3889. 10.1038/s41467-022-31212-8 35794113 PMC9259616

[bib16] Dorn JF, Zhang L, Paradis V, Edoh-Bedi D, Jusu S, Maddox PS, Maddox AS (2010) Actomyosin tube formation in polar body cytokinesis requires anillin in C. elegans. Curr Biol 20: 2046–2051. 10.1016/j.cub.2010.10.030 21055941

[bib17] Dutta S, Djabrayan NJ-V, Torquato S, Shvartsman SY, Krajnc M (2019) Self-similar dynamics of nuclear packing in the early Drosophila embryo. Biophys J 117: 743–750. 10.1016/j.bpj.2019.07.009 31378311 PMC6712419

[bib18] Elric J, Etienne-Manneville S (2014) Centrosome positioning in polarized cells: Common themes and variations. Exp Cell Res 328: 240–248. 10.1016/j.yexcr.2014.09.004 25218948

[bib19] Farhadifar R, Yu C-H, Fabig G, Wu H-Y, Stein DB, Rockman M, Müller-Reichert T, Shelley MJ, Needleman DJ (2020) Stoichiometric interactions explain spindle dynamics and scaling across 100 million years of nematode evolution. Elife 9: e55877. 10.7554/elife.55877 32966209 PMC7511230

[bib20] Freeman M, Nüsslein-Volhard C, Glover DM (1986) The dissociation of nuclear and centrosomal division in gnu, a mutation causing giant nuclei in Drosophila. Cell 46: 457–468. 10.1016/0092-8674(86)90666-5 3089628

[bib21] Goldman RD, Pollack R (1974) Uses of enucleated cells. Methods Cell Biol 8: 123–143. 10.1016/s0091-679x(08)60448-3 4366068

[bib22] Gönczy P, Pichler S, Kirkham M, Hyman AA (1999) Cytoplasmic dynein is required for distinct aspects of MTOC positioning, including centrosome separation, in the one cell stage Caenorhabditis elegans embryo. J Cell Biol 147: 135–150. 10.1083/jcb.147.1.135 10508861 PMC2164971

[bib23] Grill SW, Hyman AA (2005) Spindle positioning by cortical pulling forces. Dev Cell 8: 461–465. 10.1016/j.devcel.2005.03.014 15809029

[bib24] Grill SW, Gönczy P, Stelzer EH, Hyman AA (2001) Polarity controls forces governing asymmetric spindle positioning in the Caenorhabditis elegans embryo. Nature 409: 630–633. 10.1038/35054572 11214323

[bib25] Grill SW, Howard J, Schäffer E, Stelzer EHK, Hyman AA (2003) The distribution of active force generators controls mitotic spindle position. Science 301: 518–521. 10.1126/science.1086560 12881570

[bib26] Guo M, Ehrlicher AJ, Jensen MH, Renz M, Moore JR, Goldman RD, Lippincott-Schwartz J, Mackintosh FC, Weitz DA (2014) Probing the stochastic, motor-driven properties of the cytoplasm using force spectrum microscopy. Cell 158: 822–832. 10.1016/j.cell.2014.06.051 25126787 PMC4183065

[bib27] Hara Y, Kimura A (2009) Cell-size-dependent spindle elongation in the Caenorhabditis elegans early embryo. Curr Biol 19: 1549–1554. 10.1016/j.cub.2009.07.050 19682904

[bib28] Kamada T, Otomo K, Murata T, Nakata K, Hiruma S, Uehara R, Hasebe M, Nemoto T (2022) Low-invasive 5D visualization of mitotic progression by two-photon excitation spinning-disk confocal microscopy. Sci Rep 12: 809. 10.1038/s41598-021-04543-7 35039530 PMC8764092

[bib29] Kanesaki T, Edwards CM, Schwarz US, Grosshans J (2011) Dynamic ordering of nuclei in syncytial embryos: A quantitative analysis of the role of cytoskeletal networks. Integr Biol 3: 1112–1119. 10.1039/c1ib00059d 22001900

[bib30] Khaliullin RN, Green RA, Shi LZ, Gomez-Cavazos JS, Berns MW, Desai A, Oegema K (2018) A positive-feedback-based mechanism for constriction rate acceleration during cytokinesis in Caenorhabditis elegans. Elife 7: e36073. 10.7554/elife.36073 29963981 PMC6063732

[bib31] Khetan N, Pruliere G, Hebras C, Chenevert J, Athale CA (2021) Self-organized optimal packing of kinesin-5-driven microtubule asters scales with cell size. J Cell Sci 134: jcs257543. 10.1242/jcs.257543 34080632

[bib32] Kimura K, Kimura A (2011) Intracellular organelles mediate cytoplasmic pulling force for centrosome centration in the Caenorhabditis elegans early embryo. Proc Natl Acad Sci U S A 108: 137–142. 10.1073/pnas.1013275108 21173218 PMC3017145

[bib33] Kimura K, Kimura A (2020) Cytoplasmic streaming drifts the polarity cue and enables posteriorization of the Caenorhabditis elegans zygote at the side opposite of sperm entry. Mol Biol Cell 31: 1765–1773. 10.1091/mbc.e20-01-0058 32459552 PMC7521852

[bib34] Kimura A, Onami S (2005) Computer simulations and image processing reveal length-dependent pulling force as the primary mechanism for C. elegans male pronuclear migration. Dev Cell 8: 765–775. 10.1016/j.devcel.2005.03.007 15866166

[bib35] Kimura A, Onami S (2007) Local cortical pulling-force repression switches centrosomal centration and posterior displacement in C. elegans. J Cell Biol 179: 1347–1354. 10.1083/jcb.200706005 18158330 PMC2373484

[bib36] Kimura K, Mamane A, Sasaki T, Sato K, Takagi J, Niwayama R, Hufnagel L, Shimamoto Y, Joanny J-F, Uchida S, (2017) Endoplasmic-reticulum-mediated microtubule alignment governs cytoplasmic streaming. Nat Cell Biol 19: 399–406. 10.1038/ncb3490 28288129

[bib37] Kondo T, Kimura A (2018) Impaired chromosome segregation results in sperms with excess centrosomes in emb-27 APC6 mutant C. elegans. BioRxiv: 449538. 10.1101/449538

[bib38] Kondo T, Kimura A (2019) Choice between 1- and 2-furrow cytokinesis in Caenorhabditis elegans embryos with tripolar spindles. Mol Biol Cell 30: 2065–2075. 10.1091/mbc.e19-01-0075 30785847 PMC6727771

[bib39] Lee K-Y, Esmaeili B, Zealley B, Mishima M (2015) Direct interaction between centralspindlin and PRC1 reinforces mechanical resilience of the central spindle. Nat Commun 6: 7290. 10.1038/ncomms8290 26088160 PMC4557309

[bib40] Malone CJ, Misner L, Le Bot N, Tsai M-C, Campbell JM, Ahringer J, White JG (2003) The C. elegans hook protein, ZYG-12, mediates the essential attachment between the centrosome and nucleus. Cell 115: 825–836. 10.1016/s0092-8674(03)00985-1 14697201

[bib41] McNally KL, Martin JL, Ellefson M, McNally FJ (2010) Kinesin-dependent transport results in polarized migration of the nucleus in oocytes and inward movement of yolk granules in meiotic embryos. Dev Biol 339: 126–140. 10.1016/j.ydbio.2009.12.021 20036653 PMC2823969

[bib42] Meraldi P (2016) Centrosomes in spindle organization and chromosome segregation: A mechanistic view. Chromosome Res 24: 19–34. 10.1007/s10577-015-9508-2 26643311

[bib43] Mogilner A, Wollman R, Civelekoglu-Scholey G, Scholey J (2006) Modeling mitosis. Trends Cell Biol 16: 88–96. 10.1016/j.tcb.2005.12.007 16406522

[bib44] Munro E, Nance J, Priess JR (2004) Cortical flows powered by asymmetrical contraction transport PAR proteins to establish and maintain anterior-posterior polarity in the early C. elegans embryo. Dev Cell 7: 413–424. 10.1016/j.devcel.2004.08.001 15363415

[bib45] Nguyen PA, Groen AC, Loose M, Ishihara K, Wühr M, Field CM, Mitchison TJ (2014) Spatial organization of cytokinesis signaling reconstituted in a cell-free system. Science 346: 244–247. 10.1126/science.1256773 25301629 PMC4281018

[bib46] Nguyen PA, Field CM, Mitchison TJ (2018) Prc1E and Kif4A control microtubule organization within and between large xenopus egg asters. Mol Biol Cell 29: 304–316. 10.1091/mbc.e17-09-0540 29187577 PMC5996955

[bib47] Oegema K, Mitchison TJ (1997) Rappaport rules: Cleavage furrow induction in animal cells. Proc Natl Acad Sci U S A 94: 4817–4820. 10.1073/pnas.94.10.4817 9144146 PMC33663

[bib48] Otomo K, Hibi T, Murata T, Watanabe H, Kawakami R, Nakayama H, Hasebe M, Nemoto T (2015) Multi-point scanning two-photon excitation microscopy by utilizing a high-peak-power 1042-nm laser. Anal Sci 31: 307–313. 10.2116/analsci.31.307 25864674

[bib49] O’Connell KF (2000) The centrosome of the early C. elegans embryo: Inheritance, assembly, replication, and developmental roles. Curr Top Dev Biol 49: 365–384. 10.1016/s0070-2153(99)49018-0 11005028

[bib50] Pecreaux J, Röper JC, Kruse K, Jülicher F, Hyman AA, Grill SW, Howard J (2006) Spindle oscillations during asymmetric cell division require a threshold number of active cortical force generators. Curr Biol 16: 2111–2122. 10.1016/j.cub.2006.09.030 17084695

[bib51] Powers J, Rose DJ, Saunders A, Dunkelbarger S, Strome S, Saxton WM (2004) Loss of KLP-19 polar ejection force causes misorientation and missegregation of holocentric chromosomes. J Cell Biol 166: 991–1001. 10.1083/jcb.200403036 15452142 PMC1534123

[bib52] Rappaport R (1961) Experiments concerning the cleavage stimulus in sand dollar eggs. J Exp Zool 148: 81–89. 10.1002/jez.1401480107 14490383

[bib53] Redemann S, Pecreaux J, Goehring NW, Khairy K, Stelzer EHK, Hyman AA, Howard J (2010) Membrane invaginations reveal cortical sites that pull on mitotic spindles in one-cell C. elegans embryos. PLoS One 5: e12301. 10.1371/journal.pone.0012301 20808841 PMC2924899

[bib54] Sadler PL, Shakes DC (2000) Anucleate Caenorhabditis elegans sperm can crawl, fertilize oocytes and direct anterior-posterior polarization of the 1-cell embryo. Development 127: 355–366. 10.1242/dev.127.2.355 10603352

[bib55] Sage D, Neumann FR, Hediger F, Gasser SM, Unser M (2005) Automatic tracking of individual fluorescence particles: Application to the study of chromosome dynamics. IEEE Trans Image Process 14: 1372–1383. 10.1109/tip.2005.852787 16190472

[bib56] Saunders AM, Powers J, Strome S, Saxton WM (2007) Kinesin-5 acts as a brake in anaphase spindle elongation. Curr Biol 17: R453–R454. 10.1016/j.cub.2007.05.001 17580072 PMC2776661

[bib57] Schierenberg E, Wood WB (1985) Control of cell-cycle timing in early embryos of Caenorhabditis elegans. Dev Biol 107: 337–354. 10.1016/0012-1606(85)90316-1 3972159

[bib58] Schmidt DJ, Rose DJ, Saxton WM, Strome S (2005) Functional analysis of cytoplasmic dynein heavy chain in Caenorhabditis elegans with fast-acting temperature-sensitive mutations. Mol Biol Cell 16: 1200–1212. 10.1091/mbc.e04-06-0523 15616192 PMC551485

[bib59] Segbert C, Barkus R, Powers J, Strome S, Saxton WM, Bossinger O (2003) KLP-18, a Klp2 kinesin, is required for assembly of acentrosomal meiotic spindles in Caenorhabditis elegans. Mol Biol Cell 14: 4458–4469. 10.1091/mbc.e03-05-0283 12937278 PMC266765

[bib60] Shelton CA, Carter JC, Ellis GC, Bowerman B (1999) The nonmuscle myosin regulatory light chain gene mlc-4 is required for cytokinesis, anterior-posterior polarity, and body morphology during Caenorhabditis elegans embryogenesis. J Cell Biol 146: 439–451. 10.1083/jcb.146.2.439 10427096 PMC3206578

[bib61] Silkworth WT, Nardi IK, Paul R, Mogilner A, Cimini D (2012) Timing of centrosome separation is important for accurate chromosome segregation. Mol Biol Cell 23: 401–411. 10.1091/mbc.e11-02-0095 22130796 PMC3268720

[bib62] Tang N, Marshall WF (2012) Centrosome positioning in vertebrate development. J Cell Sci 125: 4951–4961. 10.1242/jcs.038083 23277534 PMC3533386

[bib63] Telley IA, Gáspár I, Ephrussi A, Surrey T (2012) Aster migration determines the length scale of nuclear separation in the Drosophila syncytial embryo. J Cell Biol 197: 887–895. 10.1083/jcb.201204019 22711698 PMC3384421

[bib64] Torisawa T, Kimura A (2020) The generation of dynein networks by multi-layered regulation and their implication in cell division. Front Cell Dev Biol 8: 22. 10.3389/fcell.2020.00022 32083077 PMC7004958

[bib65] von Dassow G, Schubiger G (1994) How an actin networkmight cause fountain streaming and nuclear migration in the syncytial Drosophila embryo. J Cell Biol 127: 1637–1653. 10.1083/jcb.127.6.1637 7798318 PMC2120269

[bib66] White JG, Borisy GG (1983) On the mechanisms of cytokinesis in animal cells. J Theor Biol 101: 289–316. 10.1016/0022-5193(83)90342-9 6683772

[bib67] Yesbolatova AK, Arai R, Sakaue T, Kimura A (2022) Formulation of chromatin mobility as a function of nuclear size during C. elegans embryogenesis using polymer Physics theories. Phys Rev Lett 128: 178101. 10.1103/physrevlett.128.178101 35570447

